# Temporal variation and lack of host specificity among bacterial endosymbionts of *Osedax* bone worms (Polychaeta: Siboglinidae)

**DOI:** 10.1186/1471-2148-12-189

**Published:** 2012-09-25

**Authors:** Rahel M Salathé, Robert C Vrijenhoek

**Affiliations:** 1 Monterey Bay Aquarium Research Institute, Moss Landing, CA, 95039, USA; 2 Present Address: Department of Biology and Center of Infectious Disease Dynamics, The Pennsylvania State University, Millennium Science Complex, University Park, PA, 16801, USA

**Keywords:** *Osedax*, *16S* rRNA, Ribotyping, Oceanospirillales, Endosymbionts

## Abstract

**Background:**

*Osedax* worms use a proliferative root system to extract nutrients from the bones of sunken vertebrate carcasses. The roots contain bacterial endosymbionts that contribute to the nutrition of these mouthless and gutless worms. The worms acquire these essential endosymbionts locally from the environment in which their larvae settle. Here we report on the temporal dynamics of endosymbiont diversity hosted by nine *Osedax* species sampled during a three-year investigation of an experimental whale fall at 1820-m depth in the Monterey Bay, California. The host species were identified by their unique mitochondrial *COI* haplotypes. The endosymbionts were identified by ribotyping with PCR primers specifically designed to target Oceanospirillales.

**Results:**

Thirty-two endosymbiont ribotypes associated with these worms clustered into two distinct bacterial ribospecies that together comprise a monophyletic group, mostly restricted to deep waters (>1000 m). Statistical analyses confirmed significant changes in the relative abundances of host species and the two dominant endosymbiont ribospecies during the three-year sampling period. Bone type (whale vs. cow) also had a significant effect on host species, but not on the two dominant symbiont ribospecies. No statistically significant association existed between the host species and endosymbiont ribospecies.

**Conclusions:**

Standard PCR and direct sequencing proved to be an efficient method for ribotyping the numerically dominant endosymbiont strains infecting a large sample of host individuals; however, this method did not adequately represent the frequency of mixed infections, which appears to be the rule rather than an exception for *Osedax* individuals. Through cloning and the use of experimental dilution series, we determined that minority ribotypes constituting less than 30% of a mixture would not likely be detected, leading to underestimates of the frequency of multiple infections in host individuals.

## Background

Most of the benthic marine environment, which covers nearly 70% of Earth, lacks sufficient sunlight to support photosynthesis. Although food supplies in this vast aphotic zone derive primarily from marine snow (organic detritus produced in the photic zone), dense animal communities aggregate at sites of organic enrichment resulting from debris-falls such as rotten kelp, sunken wood, and the carcasses of large marine animals [1]. Similar “oases” occur at hydrothermal vents and hydrocarbon seeps, where geochemical processes (e.g. methane and sulfide gases) support chemosynthetic microbes. Microbes also serve as epi- and endosymbionts that provide nutrition to a wide diversity of invertebrate hosts [2]. For some taxa (e.g. vesicomyid clams), the symbionts are transmitted vertically, which provides “symbiont assurance” to animal larvae that colonize newly formed habitat, but for most taxa, symbionts are acquired locally from environments in which their larvae or juveniles settle. Modes of symbiont transmission have profound consequences for evolutionary and ecological processes affecting the participants in these symbioses [3].

**Figure 1:**
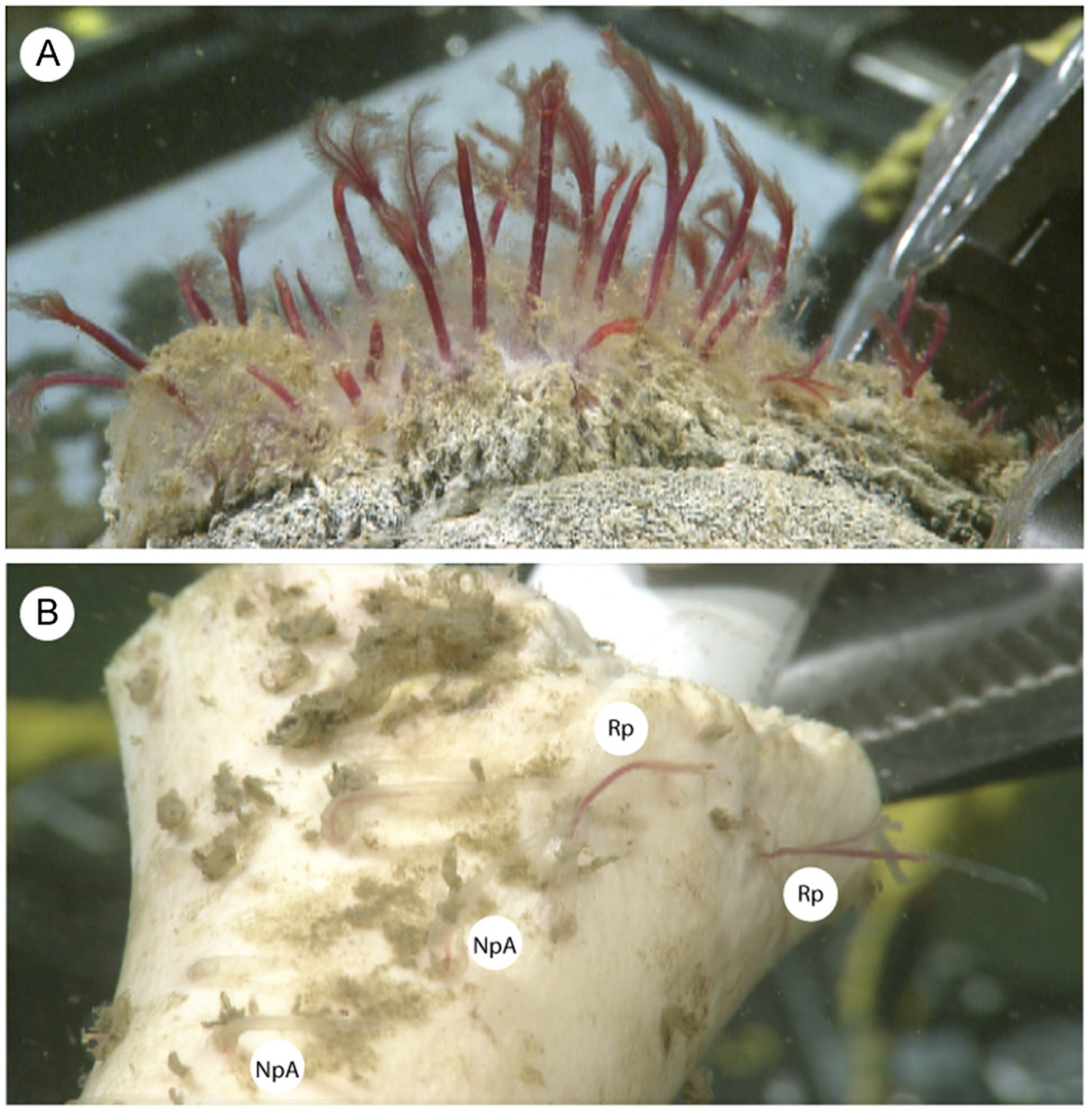
***In situ *photographs of *Osedax *on whale (A) and cow (B) bones sampled from the 1820-m locality.** Scales are similar as determined from the manipulator claw. The porous whale bone has a high density of worms composed mostly of *O. rubiplumus.* In contrast, *O. rubiplumus* and *O.* nude-palp A sparsely populated the hard surface of the cow bone.

The siboglinid tubeworms commonly found at vents, seeps and debris-falls acquire their endosymbionts environmentally [4]. Lacking mouths and digestive tracts, the adult worms rely on bacteria that live in specialized cells (bacteriocytes) concentrated in various tissue layers [5]. The family Siboglinidae encompasses four evolutionary lineages [6-8]. Vestimentiferans, monoliferans and frenulates host chemoautotrophic endosymbionts [9,10], whereas the bone-eating *Osedax* worms (Figure 1) host heterotrophic bacteria [11]. 

*Osedax* develop proliferative “roots” that penetrate sunken bones to extract organic compounds [8]. Five *Osedax* species have been named since the recent discovery of this genus [8,12-14]: *O. rubiplumus* (2004), *O. frankpressi* (2004), and *O. roseus* (2008) from Monterey Bay, California; *O. mucofloris* (2005) from the Kosterfjord, Sweden; and *O. japonicus* (2006) from Cape Nomamisaki, Japan. Twelve additional species found in the Monterey Bay were assigned morphologically descriptive “placeholder” names while they await formal descriptions [15]. Mitochondrial cytochrome-*c*-oxidase subunit-1 (*COI*) sequences provide convenient DNA-barcodes for these 17 *Osedax* species (Table 1 in reference [15]). Minimal *COI* divergence between them is 8.4%, an order-of-magnitude greater than the maximal divergence within a species (0.82%). Concordant differences exist for mitochondrial 16S rRNA and nuclear genes encoding 18S rRNA, 28S rRNA and Histone-3. Comparisons with the published DNA sequences revealed additional undescribed species from the eastern and western Pacific and the Antarctic oceans (A. Glover, personal communication). 

**Table 1:** **Submersible expeditions to recover *Osedax *samples**

			**Number *Osedax *examined**	
Date	Dive no.*	Days^†^	Whale bone	Cow bone
20-Mar-06		0	deployed	
23-May-06	T990	64	9	deployed
24-Oct-06	T1048	218	24	9
10-Jan-07	T1071	296	21	0
15-Aug-07	T1119	513	22	14
18-Dec-07	T1163	638	20	14
10-Mar-09	DR12	1086	27	6

* ROV *Tiburon* dives preceded by “T”; and ROV *Doc Ricketts* dives preceded by “DR”.

^†^ Days since deployment of whale-1820 carcass.

Ribotyping (16S rRNA sequencing) studies identified a diverse assemblage of primary and secondary microbes associated with *Osedax* and the sediments surrounding whale bones [11,12,16-18]. The primary endosymbionts, Gammaproteobacteria related to *Neptunomonas* and belonging to the order Oceanospirillales, live in bacteriocytes located in the interior tissue that surrounds the ovisac and proliferative roots [5,13,16,19]. *Osedax* eggs and pre-settling larvae are aposymbiotic [20]. They acquire the primary endosymbionts following settlement, but the mechanism and timing of infections remain unclear [5,18]. The aposymbiotic larvae of another siboglinid tubeworm, *Riftia pachyptila*, are infected transdermally by thiotrophic bacteria during a narrow window of development that precedes metamorphosis to a juvenile stage [21]. *Osedax*, on the other hand, are hypothesized to acquire endosymbionts repeatedly as individual roots proliferate through sunken bones [18]; consequently, multiple ribotypes can occur within individual bacteriocytes, and frequencies of the ribotypes may vary among the lobules of a worm’s ovisac and root system. Juvenile and adult stages of *O. frankpressi* collected several months apart from the same whale carcass hosted different symbiont strains [16]. Compared with vestimentiferans, the primary endosymbionts associated with *Osedax* are more dynamic and diverse.

To assess the temporal dynamics of endosymbiont diversity in *Osedax*, we conducted a three-year time series analysis of Oceanospirillales bacteria from an experimental whale-fall deployed during March 2006 at 1820 m depth in Monterey Bay, California. Cow bones were also deployed at the site, and samples were obtained with robotic submarines six times during this three-year period (Table 1). *Osedax* species sampled from whale and cow bones were identified by their unique *COI* barcodes [15]. The associated bacteria were characterized by ribotyping, which was facilitated by employing PCR primers specifically designed to amplify the *16S* rRNA sequences from these Oceanospirillales bacteria [22]. The sequence traces obtained with direct sequencing of PCR products tend to reveal only the majority targets in complex bacterial mixtures. To better assess within-host diversity of these bacterial strains, we examined clone-libraries generated from a subset of individuals. Furthermore we tested the reliability of standard PCR reactions in detecting multiple infections by examining dilution series constructed from mixed ribotypes. As essentially all individuals proved to be multiply infected, we also examined the potential for compartmentalization, i.e. physical separation of the different endosymbiont strains, among different anatomical parts of a host individual. Finally, we conducted two-way contingency tests to assess host-symbiont specificity and the impact of environmental factors on the distribution of symbiont ribotypes. Throughout this publication, we use the terms “ribotype” to denote bacterial strains marked by a distinct *16S* sequence and “ribospecies” to denote a grouping of ribotypes that share ≥ 97% sequence similarity sensu [23,24].

## Methods

### Sample collection

Bones colonized by *Osedax* (Figure 1) were sampled from the carcass of a juvenile gray whale deployed on 20 March 2006 at a depth of 1820 m in the Monterey Submarine Canyon, CA (36.772° N and 122.083° W). Subsequent monitoring of the whale-fall community at this site has been described [25,26]. Cow bones were deployed on 23 May 2006 approximately 10 m away from the whale carcass [27]. We obtained the present samples during six R/V *Western Flyer* expeditions (Table 1) that employed the remotely operated vehicles (ROVs) *Tiburon* and *Doc Ricketts* operated by the Monterey Bay Aquarium Research Institute (MBARI). Whale bones were recovered during all six expeditions and cow bones were recovered during expeditions 2–6. All bones were sampled with robotic manipulators and placed in seawater-filled containers that were closed to maintain near-bottom temperatures (mean = 2.3°C) during the ascent. A sediment sample was taken with a 9 cm-wide push-core next to a whale bone sampled during dive T1163. At the surface, the bones were stored in cold filtered seawater (4°C) before removal of the embedded *Osedax* worms. Individual worms were stored in separate cryovials containing 95% ethanol or frozen at −80°C.

**Figure 2:**
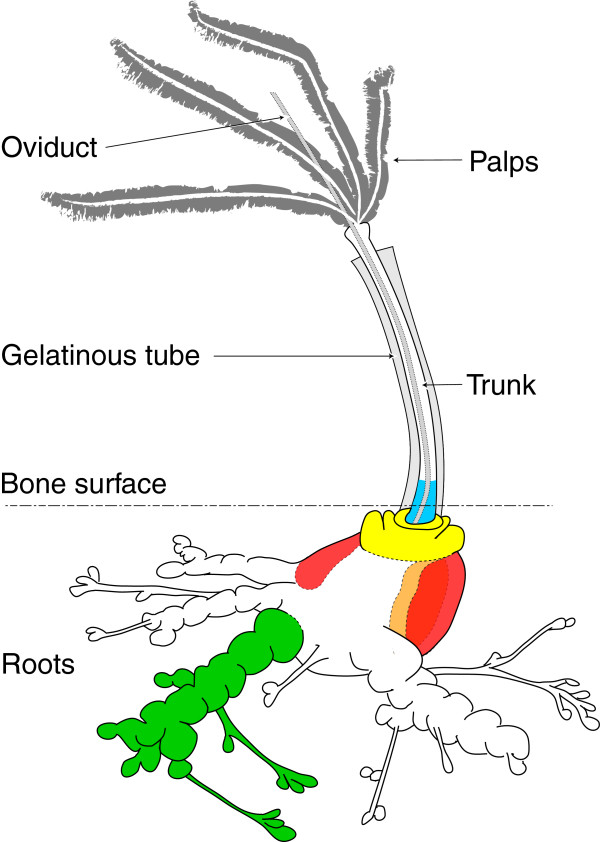
**Schematic of *Osedax *individual showing the tissues examined for endosymbiont compartmentalization: blue = basal trunk; yellow = anterior ovisac; red = outer ovisac sheath; orange = inner ovisac tissue; and green = posterior ovisac/ root.** Drawing is based on a watercolor illustration by Howard Hamon of the South Australia Museum, Adelaide.

We examined 162 *Osedax* worms for the present analyses. Root, trunk and palp tissues (Figure 2) were dissected from each individual, if possible, and stored separately for subsequent analyses. To test for possible compartmentalization of the symbiont infections within host individuals (i.e. physical separation of multiple infections), we dissected basal trunk tissue, anterior ovisac, posterior ovisac/root, outer ovisac sheath, and inner ovisac tissues from 21 of the larger specimens (7 worms from dive T1119, and 14 worms from dive DR12). Separate lobes of ovisac tissues were examined individually from several of the larger worms.

### General phylogenetic and statistical methods

Sequence alignments were conducted with ClustalX 2.0.12 [28], and edited in MacClade 4.08 [29]. Analysis of DNA sequence diversity was conducted with MEGA5 [30]. Appropriate DNA substitution models were determined with JModeltest v. 1.0.1 [31,32]. The model with the lowest Bayesian Information Criterion (BIC score) was chosen for phylogenetic analyses conducted with Mr. Bayes v. 3.1.3 [33,34]. Each analysis was conducted as six chains for 5.1 × 10^6^ generations. Print and sample frequencies were 1 000 generations, and the burn-in was the first 100 samples. Analyses were repeated five times and the resulting data were visualized using Tracer v. 1.3 [35] to determine the appropriate burn-in period and ensure data had reached convergence. Trees were visualized using FigTree v. 1.3.1 [36]. A parsimony network of *16S* sequences was constructed with the program TCS v. 1.18 [37]. Two-way contingency tests of independence between endosymbiont ribospecies, host species, time of sampling, and type of bone were conducted with JMP v. 7.02 software [38].

### Analysis of host COI sequences

*Osedax* individuals were identified by their unique *COI* barcodes following previously described procedures [15]. Briefly, we used the DNeasy kit (Qiagen, Valencia, CA) to extract DNA. PCR was conducted with AmpliTaq Gold (Applied Biosystems Inc., Foster City, CA, USA) and *COI* primers developed for siboglinid worms [39] to amplify approximately 1 200 bp of sequence*.* PCR parameters were as follows: initial denaturation at 95°C/10 min, 35 cycles (94°C/1 min, 55°C/1 min, and 72°C/ 1 min), and final extension at 72°C/7 min. *COI* amplicons were diluted in 50 μl sterile H_2_O and cleaned with Multiscreen HTS PCR 96 filter plates (Millipore Corp., Billerica, MA, USA). Sequencing reactions were conducted with the same primers, and the resulting sequences were analyzed bidirectionally on an ABI 3100 using BigDye terminator v.3.1 chemistry (Applied Biosystems Inc., Foster City, CA, USA). *Osedax* sequences new to this study were deposited in GenBank (acc. nos. JX280608 - 613) and compared with published sequences [GenBank acc. nos. in references: 8, 12, 13, 15, 25].

### Analysis of symbiont 16S sequences

**Figure 3:**
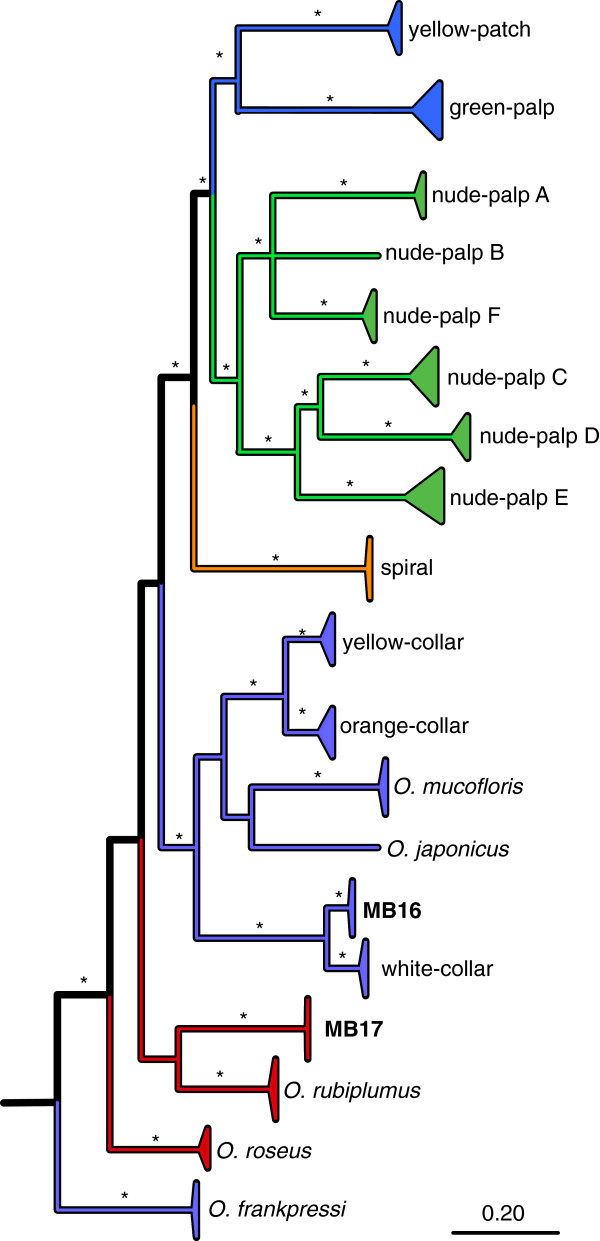
**Bayesian phylogenetic relationships of mitochondrial *COI *in 19 Operational Taxonomic Units (OTUs) of *Osedax *boneworms.** Two OTUs are new to this study (MB16 and MB17). The triangles represent the maximum depth of the presently known sequence diversity in each OTU. Asterisks (*) on nodes and branches represent Bayesian posterior probability (BPP) values ≥0.99. Branches are colored to be consistent with previously published groupings [15].

New 16S sequences were obtained with primers specifically designed to amplify a 672 bp fragment from *Oceanospirillales* bacteria associated with *Osedax*[22]: 435 F: 5'-CAGCWGTGAGGAAAGGTT-3', and 1213R: 5'-TGTGTAGCCCAACTCG-3'. PCR was conducted with HotStartTaq (Qiagen, Valencia, CA) according to the following parameters: initial denaturation at 95°C/10 min, 30 cycles (94°C/1 min, 54°C/1 min, and 72°C/1 min), and final extension at 72°C/10 min. If initial concentrations of target DNA were small, we added two to five PCR cycles. Purification and sequencing of amplicons were conducted as before with *COI*. PCR reactions were repeated in cases when chromatograms of the sequence traces appeared to involve mixed infections by multiple symbiont strains, and to verify singletons, i.e. ribotypes found only once in this study. Due to sequence ambiguities in close proximity of the forward and reverse primers, the sequences were trimmed at these ends and final segments of 672 bp length used for the analysis. *Oceanospirillales* sequences new to this study were deposited in GenBank (acc. nos. JX280614–661) and compared with published ribotypes [GenBank acc. nos. in Figure 3; references: 13, 16–18]. 

### Assessing mixed infections

Sequence traces were used to designate individual worms as multiply infected if the secondary peak at a polymorphic nucleotide site was at least half of the height of the primary peak. We did not consider lesser peaks, as they were confounded with background variation. Potentially mixed sequence traces were verified by re-amplification and sequencing. We resolved the phases of mixed symbiont ribotypes in the following ways. Mixtures involving ambiguities at single nucleotide positions (e.g., Y = T or C at position 663) could be manually decomposed into alternative ribotypes. If ambiguities occurred at multiple positions (e.g., R = A or G at position 366 and Y at position 663), we inferred phases of the constituent ribotypes by generating clone libraries with the Invitrogen Topo TA cloning kit (Invitrogen, Carlsbad, CA). Singleton ribotypes in the libraries that were not repeated were treated as cloning artifacts and excluded from subsequent analyses, as were the remaining unresolved ambiguities.

Dilution series were used to determine whether PCR “dropouts” of minority ribotypes due to low quantities of target DNA occurred in mixed infections. Various ratios (e.g., 0:100, 10:90. 20:80, … ,100:0) were generated from two pairs of cloned *16S* amplicons, and one pair of un-cloned samples that differed at one or more nucleotide positions. Each dilution series was replicated three times. PCR reactions were conducted with the known mixtures, and amplicons were sequenced as previously described.

## Results

### Host diversity

Based on previously published *COI* sequences [15]*,* two host taxa, MB16 and MB17, were new to this study (Figure 3). *Osedax* MB16 is related to white-collar (sequence divergence, *D* = 7.4%), whereas *Osedax* MB17’s is most closely related to *O. rubiplumus* (*D* = 20.3%). Altogether, the present sample of 162 *Osedax* worms contained nine host species in the following order of abundance: *O. rubiplumus* (*n =* 76), *O. frankpressi* (*n =* 34), green-palp (*n =* 14), nude-palp A (*n =* 12), nude-palp D (*n =* 6), *O. roseus* (*n =* 3), MB17 (*n =* 3), MB16 (*n =* 2); nude-palp C (*n =* 1). Eleven very small worms did not provide reliable *COI* sequences after repeated attempts and remained unidentified. For subsequent statistical analyses, they were nested in the category “other” (Table 1).

### Symbiont diversity

The symbiont–specific primers amplified 48 distinct *16S* ribotypes in the present samples (GenBank acc. nos. JX280614–661). Thirty-two ribotypes were revealed with PCR and direct sequencing from symbiont-bearing tissues, and 16 were revealed in clone libraries. These 672-bp *16S* sequences exhibited 149 polymorphic sites (*S*). The mean number of nucleotide substitutions between pairs of ribotypes was 33.8. For these 48 ribotypes, the mean nucleotide diversity per site (π) was 0.05021 and the normalized diversity (θ_S_) was 0.05214 per site.

**Figure 4:**
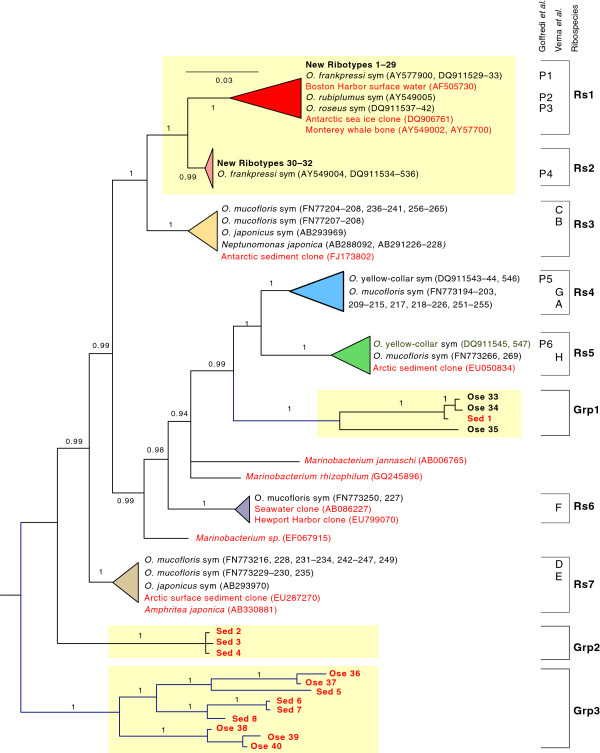
**Bayesian phylogenetic analysis of ribotype sequences from Oceanospirillales bacteria associated with *Osedax *bone worms (black font) and environmental samples (red font).** The yellow highlighted branches include sequences that are new to this study. Closely related endosymbiont ribotypes with genetic distances ≤ 3% were assembled into triangles and designated as ribospecies Rs1–Rs7. For reference, the phylotype designations of Goffredi *et al.*[11] (P1–P6) and Verna *et al.*[18] (A–H) are nested within the new ribospecies designations. Groupings of recovered ribotypes that did not cluster with endosymbiont lineages are indicated as Grp1-Grp3). The numbers along branches represent Bayesian posterior probability distributions (PPDs). The branches with PPDs <0.94 were collapsed into polytomies (e.g., *M. jannaschi* and *M. rhizophilum*).

We conducted a Bayesian phylogenetic analysis of the 48 new ribotypes (highlighted in yellow, Figure 4) in conjunction with previously published *16S* sequences, mostly from whale-fall samples. Symbiont ribotypes that differed by ≤ 3% were clustered into seven distinct ribospecies (Rs1–Rs7, Table 2) that were clearly distinguished as well-supported clades in the Bayesian tree (Posterior Probabilities ≥0.99). Ribospecies Rs3–Rs7 were not found among the present samples from the 1820-m locality. Rs4 and Rs5 were previously identified from shallower whale-falls (383 m) in Monterey Bay and Sweden, and Rs3, Rs6 and Rs7 were previously identified from shallow whale-falls off Sweden (25–125 m) and Japan (~200 m). Thirty-two of the new ribotypes obtained with PCR and direct sequencing from *Osedax* tissues fell into ribospecies Rs1 and Rs2. Sixteen of the new ribotypes were identified in clone libraries derived from *Osedax* tissues (Ose 33–40) and a sediment sample (Sed 1–8). They were distributed in three regions of the tree and were not related to the primary *Osedax* endosymbionts. Because the tree is unrooted, we cannot infer ancestral states. 

**Table 2:** **Sequence divergence within (bold italics, on diagonal) and between (lower triangle) major bacterial clades illustrated in the Bayesian phylogenetic analysis (Figure**4**)**

	**Rs1**	**Rs2**	**Rs3**	**Rs4**	**Rs5**	**Rs6**	**Rs7**	**Grp2**	**Grp1**	**Grp3**
Rs1	***0.0102***									
Rs2	0.0352	***0.0020***								
Rs3	0.0729	0.0512	***0.0031***							
Rs4	0.1203	0.0862	0.0892	***0.0182***						
Rs5	0.1377	0.1206	0.0947	0.0737	***0.0158***					
Rs6	0.1153	0.0804	0.0679	0.0869	0.1119	***n/c***				
Rs7	0.0764	0.0681	0.0542	0.1025	0.1046	0.0635	***0.0095***			
Grp2	0.1590	0.1493	0.1561	0.1668	0.1788	0.1621	0.1117	***0.0010***		
Grp1	0.1901	0.1924	0.1779	0.1665	0.1689	0.1650	0.1862	0.2136	***0.0630***	
Grp3	0.2220	0.2070	0.2011	0.2167	0.2007	0.2273	0.1927	0.2301	0.2663	***0.1090***

### Host, substrate and temporal components of symbiont diversity and abundance

**Figure 5:**
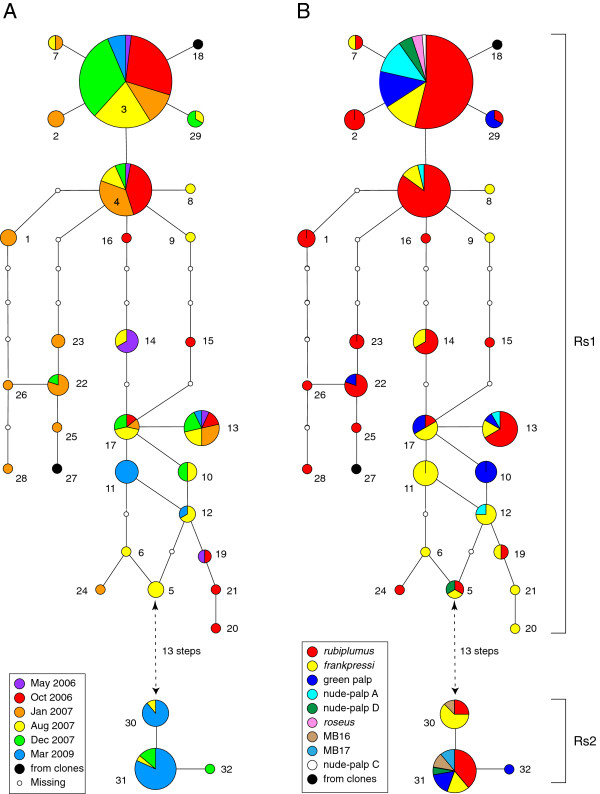
**Parsimony networks for endosymbiont ribotypes 1–32: A. Colored pie-slices indicate the proportion of ribotypes assigned to: (A) sample periods; and (B) host species (unidentified hosts excluded).** Sizes of the pies scaled to reflect ribotype frequencies. Connecting lines indicate a single mutational step. Empty dots indicate inferred ribotypes that were not sampled in this study.

The 32 ribotypes obtained by direct sequencing from the *Osedax* tissues fell into ribospecies Rs1 (ribotypes 1–29) and Rs2 (ribotypes 30–32), which differed minimally by 13 nucleotide substitutions with a mean sequence divergence of 3.52%. A statistical parsimony network (Figure 5) clearly reveals that Rs1 was more diverse and abundant in the present samples. Half of the Rs1 ribotypes (52%) were singletons that were verified with second PCRs from the original tissue extracts. One of the three Rs2 ribotypes was a singleton.

Frequencies of Rs1 and Rs2 shifted during this time-series (Figure 5A). A two-way contingency test of ribospecies frequencies against sample dates revealed a highly significant association (Table 3A). Rs1 was overrepresented in the early samples through Dec-07 and Rs2 was significantly overrepresented in the Mar-09 sample. Frequencies of the *Osedax* host species also changed significantly during this period (Table 3C). *O. Rubiplumus* was overrepresented in the first two samples and it became increasingly scarce after Aug-07. *O. frankpressi* was not abundant until Aug-07. *O.* green-palp and *O.* nude-palp-A were only abundant in the Dec-07 sample. The remaining five species were rare and lumped into the category “other” to avoid possible statistical artifacts due to “sampling zeros” [40]. Although the hosts and endosymbionts both varied significantly with time, they exhibited no association with one another (Figure 5B; Table 3D). Rs2 clearly dominated in all the host species. Frequencies of the two ribospecies were independent of whale or cow bone substrates (Table 3B), but frequencies of the host species were associated with the type of bone (Table 3E). *O. rubiplumus* and *O frankpressi* densely populated whale bones, but they were rare on the cow bones (Figure 1). These larger worms may have obscured the presence of small and relatively transparent species, such as *O.* green-palp and O. nude-palp-A, on the densely populated whale bones. The smaller worms were more readily observed and sampled from the sparsely populated cow bones (Figure 1B). 

**Table 3:** Two-way contingency tests involving symbiont ribospecies, host species, bone substrates and dive dates

**A. Symbiont ribospecies by date**											
Date	Rs1		Rs2		Total						
Oct-06	31	*27.0*	1	*5.0*	32						
Jan-07	18	*16.0*	1	*3.0*	19						
Aug-07	35	*29.5*	0	*5.5*	35						
Dec-07	32	*28.7*	2	*5.3*	34						
Mar-09	13	*27.8*	20	*5.2*	33						
Total	129		24		153						
	*G* = 56.736; *df* = 5; *P* < 0.0001^*^										
**B. Symbiont ribospecies by bone type**											
Bone	Rs1		Rs2		Total						
Whale	94	*93.6*	17	*17.4*	111						
Cow	35	*35.4*	7	*6.6*	42						
Total	129		24		153						
	*G* = 0.042; *df* = 1; *P* = 0.838										
**C. Host species by date**											
Date	*rubiplumus*		*frankpressi*		green-palp		nude-palp A		other		Total
Oct-06	22	*14.0*	1	*7.1*	1	*2.7*	0	*2.5*	8	*5.6*	32
Jan-07	19	*8.3*	0	*4.2*	0	*1.6*	0	*1.5*	0	*3.4*	19
Aug-07	10	*15.3*	14	*7.8*	0	*3.0*	2	*2.7*	9	*6.2*	35
Dec-07	9	*14.9*	0	*7.6*	12	*2.9*	10	*2.7*	3	*6.0*	34
Mar-09	7	*14.5*	19	*7.3*	0	*2.8*	0	*2.6*	7	*5.8*	33
Total	67		34		13		12		27		153
	*G* = 140.298; *df* = 16; *P* < 0.0001										
**D. Host species by symbiont ribospecies**											
Ribo-species	*rubiplumus*		*frankpressi*		green-palp		nude-palp A		other		Total
Rs1	8	*10.5*	7	*5.3*	2	*2.0*	1	*1.9*	6	*4.2*	24
Rs2	59	*56.5*	27	*28.7*	11	*11.0*	11	*10.1*	21	*22.8*	129
Total	67		34		13		12		27		153
	*G* = 2.702; *df* = 4; *P* < 0.6088										
**E. Host species by bone type**											
Bone	*rubiplumus*		*frankpressi*		green-palp		nude-palp A		other		Total
Whale	63	*48.6*	32	*24.7*	0	*9.4*	10	*8.7*	6	*19.6*	111
Cow	4	*18.4*	2	*9.3*	13	*3.6*	2	*3.3*	21	*7.4*	42
Total	67		34		13		12		27		153
	*G* = 94.900; *df* = 4; *P* < 0.0001										

^*^G = log likelihood ratio*; df* = degrees of freedom; and *P* = probability.

### Assessing multiple symbiont infections

PCR and direct sequencing occasionally produced ambiguous sequence traces that suggested mixed symbiont infections within individual worms. To verify the presence of mixtures, we generated clone libraries from a subsample of 15 worms. Depending on the diversity observed, 12 to 47 clones were examined from each worm. Each of the 15 worms exhibited mixed infections involving at least two to nine distinct ribotypes. Although PCR and direct sequencing never identified a worm that simultaneously hosted ribospecies Rs1 and Rs2, the clone libraries identified one worm from a cow bone sample (T1163.cb1) that had both.

To determine the detection limits for multiple infections with PCR and direct sequencing, we generated dilution series involving mixtures of pure ribotypes from cloned Rs1 sequences and from mixtures involving different worms that hosted the Rs1 (worm F) and Rs2 (worm E) ribospecies (Table 4). Limits of detection were affected by the position of single nucleotide polymorphisms (SNPs) along the length of the 672 bp amplicons. Based on this procedure we determined that minority ribotypes constituting less than 30% of the sequences in a mixture would not likely be detected by standard PCR.

**Table 4:** Detection of nucleotide composition at sites that are polymorphic in paired samples

		**Nucl.**	**Dilution ratio**										
Mixture	Rep.	position	0:100	10:90	20:80	30:70	40:60	50:50	60:40	70:30	80:20	90:10	100:0
A x B	1	666	C	C	C	**Y**	**Y**	**Y**	**Y**	T	T	T	T
	2		C	C	C	**Y**	**Y**	**Y**	**Y**	T	T	T	T
	3		C	C	C	**Y**	**Y**	**Y**	**Y**	T	T	T	T
C x D	1	366	A	A	**R**	**R**	**R**	**R**	**R**	G	G	G	G
	2		A	A	**R**	**R**	**R**	**R**	G	G	G	G	G
	3		A	A	**R**	**R**	**R**	**R**	G	G	G	G	G
E x F	1	120	T	T	T	**W**	**W**	**W**	**W**	**W**	A	A	A
	2		T	T	T	**W**	**W**	**W**	**W**	**W**	A	A	A
	3		T	T	T	**W**	**W**	**W**	**W**	**W**	A	A	A
	1	363	A	A	A	**R**	**R**	**R**	**R**	**R**	G	G	G
	2		A	A	A	**R**	**R**	**R**	**R**	**R**	G	G	G
	3		A	A	A	**R**	**R**	**R**	**R**	**R**	G	G	G
	1	660	G	G	G	G	**S**	**S**	**S**	**S**	C	C	C
	2		G	G	G	G	**S**	**S**	**S**	**S**	C	C	C
	3		G	G	G	G	**S**	**S**	**S**	**S**	C	C	C

Dilution series were created from mixtures of bacterial *16S* amplicons obtained from the following *Osedax* samples: (A) T1048.115 clone E6; (B) T1048.115 clone D11; (C) T1071.13 clone D4; (D) T1071.13 clone C6; (E) worm T1071.35; (F) worm T1048.110. Each mixture was replicated three times. Mixtures detected by sequencing software are marked in boldface.

### Symbiont compartmentalization

To assess whether the different constituents of multiple infections were physically separated among various body parts of host individuals, we examined tissue subsamples from 21 worms that were large enough to allow isolation of three or more distinct tissues (Table 5). *16S* sequences were amplified from all the tissue types examined. Three out of seven worms from dive T1119 and five out of 14 worms from dive DR12 generated symbiont sequences from green colored posterior trunk tissues. Several of the worms hosted multiple infections, e.g., worm T1119.2 hosted ribotypes Rs1.3 and Rs1.29, worm T1119.4 hosted ribotypes Rs1.13 and Rs1.30, worm DR12.1.1 hosted Rs1.9 and Rs1.10, and worm DR12.1.10 hosted Rs1.11 and Rs1.12, which corresponds to 19% of infections involving multiple strains. Two host individuals (DR12.1.1 and T1119.2) exhibited signs of compartmentalization of different symbiont strains in ovisac sheath and inner ovisac tissues, and two other individuals (T1119.4 and DR12.1.10) simultaneously hosted two different ribotypes in their trunk tissues.

**Table 5:** Ribotype amplifications from different host tissues of seven worms from Dive T1119 and 14 worms from Dive DR12

			**Ribotypes identified in tissue compartments**				
Dive no.	Bone no.	Worm no.	Basaltrunk	Anterior ovisac	Outer ovisac	Interior ovisac	Posterior ovisac/root
T1119		1	na^*^	3	3	3	3
		2	na	na	3	3, 3 + 29^**^	3
		4	13 + 30	13	13	13	13
		5	3	3	3	na	3
		7	3	3	3	3, 3	3
		8	na	3	3	3	na
		9	na	na	na	na	3
DR12	1	1	na	-	10	9	-
	1	2	-	-	10	10	10
	1	5	10	10	-	10	-
	1	10	11 + 12	11	-	11	12
	1	11	na	3	-	3, 3, 3	3
	1	14	na	-	11	11	11
	1	15	na	-	-	3, 3, na	3
	1	16	na	9	-	na	9
	2	1	na	10	-	-	10
	2	2	na	10	-	-	10
	2	3	9	9	-	-	9
	2	7	na	3	-	3	3
	2	10	9	9	-	9	9
	2	11	13	13	-	-	13

^*^ na: no amplification from tissue, i.e. no signal for symbiont presence.

^**^ The plus signs denote mixed ribotype infections. Commas separate ribotypes obtained from separate lobes of ovisac tissue.

^†^ dashes indicate tissues that were not available from a host individual.

## Discussion

### *Osedax* hosts

Nine *Osedax* lineages were sampled during this study, including three named species (*O. rubiplumus*, *O. frankpressi*, and *O. roseus*), four with placeholder names (*O.* green-palp, *O.* nude-palp A, *O.* nude-palp D, and *O.* nude-palp C), and two (MB16 and MB17) reported here for the first time. Comparative analyses of *COI* sequences between the undescribed lineages and known *Osedax* species worldwide indicate that MB16 and MB17 deserve recognition as species. Ongoing studies involving a suite of nuclear and mitochondrial genes previously employed in *Osedax* systematics corroborate the distinct nature of MB16 and MB17 (S. Johnson, unpublished).

*Osedax* communities exhibit successional changes as bones decompose [25,26]. *O. rubiplumus* is an early colonizer that develops relatively shallow filamentous roots. It densely populated whale bones at the 2893-m and 1820-m Monterey whale-falls, and then was replaced by *O. frankpressi*, which produces a robust lobular root system that penetrates more deeply into bones. *O.* spiral, an unnamed species found at the 2893 m whale-fall, appeared even later and occupied bone fragments that were buried in sediments [15]. In the present study, *O. rubiplumus* and *O. frankpressi* exhibited a highly significant association with the time of sampling (*G* = 57.915; *df* = 4; *P* < 0.00101). *O.* green-palp was disproportionally represented in the Dec-07 sample and rare at other times. The other host species were relatively infrequent throughout the study period, providing no foundation for assessing changes in their abundance.

The type of bone appears to affect frequencies of the host species (Figure 1). Whale bones deployed at the 2893-m and 1820-m localities were densely populated by *O. rubiplumus* and *O. frankpressi*, whereas cow bones, which have less porous surfaces (Figure 1B), were sparsely populated [27]. The smaller transparent species appeared to be more frequent on cow bones, but this may be due to the ease of seeing smaller worms when the larger species are scarce. Nonetheless, the bones of bovids do not naturally occur in marine environments, unless they are washed in during terrestrial floods or dumped as organic galley waste from passing ships [41]. Differences may exist among *Osedax* species in their natural utilization of bones from various vertebrate taxa, but a number of species can grow and reproduce on a range of bones including those from teleosts [22]. More recent experimental deployments of sea lion, elephant seal, turkey and sea turtle bones in Monterey Bay have also been colonized by one or more species of *Osedax* (Vrijenhoek, unpublished).

### Oceanospirillales endosymbionts

The endosymbionts associated with these worms clustered into seven distinct clades (Rs1–Rs7) that we treat as ribospecies (Figure 4). The ribospecies concept has limitations for studies of microbial ecology and community structure, because it fails to consider functional genomic characteristics that might distinguish evolutionary lineages [24,42,43]. Nonetheless, it provides a useful method for reducing hierarchically lower-level molecular diversity into manageable clusters of related genotypes. Ribospecies Rs1 and Rs2 comprise a well-supported monophyletic assemblage that except for two environmental samples is restricted to deep waters. Rs1 and Rs2 were the primary endosymbionts in *Osedax* that grew on bones deployed at the 1820-m site. To date, we have not found these ribospecies at whale-falls shallower than 1000 m in Monterey Bay [16].

In contrast, ribospecies Rs3 through Rs7 comprise a paraphyletic assemblage that has only been reported from samples obtained at depths shallower than 500 m. Rs3 should adopt the name *Neptunomonas japonica* for a free-living member cultured from whale-fall sediments off Kagashima, Japan [17]. Several of these shallow ribospecies have widespread distributions, and their paraphyletic assemblage is interspersed with free-living lineages including several *Marinobacterium* species, Sed1–8 ribotypes, and possibly the Ose33–40 ribotypes.

Although frequencies of ribospecies Rs1 and Rs2 varied independently of the *Osedax* host species, the endosymbiont population changed significantly with time. Goffredi *et al.*[16] first suggested that *Osedax* endosymbionts varied with time and the developmental stages of *Osedax* on whale bones. Similarly, an analysis of *O. mucofloris* endosymbionts from Minke whale carcasses found that time could explain about 31% of the variance in ribotype diversity of the endosymbionts, leading Verna *et al.*[18] to consider three hypotheses. From the present data, we can exclude their first hypothesis (*i*): “it was an artefact caused by the low number of individuals available for each sampling group.” Our sample of host individuals was equitably represented across the entire time series (Table 1C). Furthermore, by grouping the 32 ribotypes into ribospecies Rs1 and Rs2, we avoided a potential statistical artifact due to sampling zeros, a consequence of small sample sizes [40]. Their second hypothesis seems plausible in the present case (*ii*): “the free-living population from which the endosymbionts were taken up varied over time, either randomly or because of environmental changes in the chemical and biological milieu at the whale-fall.” The type of bone, whale or cow, did not affect ribospecies frequencies, but the endosymbionts are mostly located within the hosts’ interior tissues and may not contact the bones directly. On the other hand, sediments surrounding the bones and supporting free-living infectious stages of these bacteria might play a role in this variation. Goffredi *et al.*[44] reported that ribotype composition of the archaeal community in sediments around the 2893-m Monterey whale-fall varied considerably in time with changes in methane and sulfide concentrations. A similar analysis of sediment samples for free-living components of the endosymbiont population is warranted. Verna *et al*.’s [18] third hypothesis seems less likely (iii): “choice of endosymbionts by host individuals varied over time either stochastically or because of specific selection processes driven by factors such as changes in the host’s environment.” Hypothesis *iii* requires the host to express a bacterial surveillance system that changes with time or conditions. If various host species with different morphologies, successional preferences, and potentially physiological characteristics do not appear to discriminate among the ribotypes of Rs1 and Rs2, it is difficult to imagine that any one species would exhibit changing “preferences” over time. For now, the simplest hypothesis is that acquisition of endosymbionts by *Osedax* is opportunistic, reflecting the changing composition of Oceanospirillales ribospecies in the local environment, but it is not random. Acquisition of the primary endosymbionts is clearly constrained to a subset of ribospecies that does not include the diverse paraphyletic assemblage of lineages found in the sediment sample (Sed1–8). Although related lineages (Ose33–40) were found in clone libraries from *Osedax* tissues, we suspect that these minority strains might be incidental infections that could not grow to high densities in the host’s tissues. Alternatively, they might be incidental epibionts because the worms could not be cleanly separated from associated bone and sediment contamination. Further experiments involving fluorescence in situ hybridization (FISH) with strain-specific probes are required to test whether epibionts are involved.

### Mixed infections, tissue compartmentalization, and endosymbiont acquisition

Mixed endosymbiont infections can segregate among different tissue compartments of individual *O. mucofloris* worms [18]. Direct sequencing of *16S* amplicons also revealed mixed sequence traces in a number of the *Osedax* individuals that we examined. *16S* clone libraries from a subset of worms revealed that each individual is likely to host multiple infections, with two to nine ribotypes per worm. Electron microscopy has revealed the presence of endosymbiont bacteria in root and ovisac tissues, within the lumen of multicellular glands associated with the trunk epidermis, but not the epidermis itself, where contact to bone material takes place [19]. We amplified and sequenced endosymbiotic ribotypes from root, different compartments of ovisac, and trunk (Figure 2, Table 5). Most of the worms (38.09%) housed endosymbionts in root tissues; 88.24% housed them in the anterior ovisac; 90.00% in the outer ovisac; and 78.95% in the inner ovisac. Some trunk and ovisac tissues housed multiple endosymbiont strains, but root tissues typically housed a single dominant strain. Katz *et al.*[19] reported that root tissues contain intact and dividing (i.e. active) endosymbionts. Proliferating root tissues appear to be sites of bone degradation, nutrient uptake and transport, whereas anterior portions of roots and the ovisac contain endosymbionts that appear to be degrading. How *Osedax* acquire their symbionts from the environment still remains unclear. Borrowing from a model developed for the vestimentiferan *Riftia pachyptila*, and evidence from FISH microscopy, Verna *et al.*[18] suggest that free-living bacteria in whale bones are translocated through the epidermis of proliferating *Osedax* roots. In contrast to *Riftia*, however, there appear to be repeated events of endosymbiont acquisition in *Osedax*.

## Conclusions

Our analysis of cloned sequences from *16S* rRNA libraries indicated that we should expect essentially every *Osedax* individual to be infected with multiple ribotype strains of *Oceanospirillales* bacteria. In contrast, direct sequencing following PCR from *Osedax* tissues only revealed majority ribotypes infecting a particular tissue sample. PCR targets with low copy-number (≤ 30%) could not be detected with direct sequencing. The two methods present different costs and benefits, however. Time and funding often preclude the generation and screening of clone libraries from large time-series or numerous geographical samples. Nonetheless, cloning clearly provides a useful method for identifying the within-individual component of endosymbiont diversity. Though it sacrifices the within-individual component, and the level of multiple infections will most likely be underestimated, direct sequencing still provides an efficient method for screening the between-individual component of symbiont diversity in large population samples. New high-throughput screening methods provide a similar advantage for screening within-individual components of variance as does traditional cloning see for example [45]. And so as costs for these new-generation methods continue to decline the tradeoffs involved in obtaining the within- and among-individual components of diversity should soon disappear.

## Competing interests

The authors declare that they have no competing interests.

## Authors’ contributions

RS and RV designed the study and drafted the paper. RS participated in the sampling and dissection of *Osedax* hosts. She conducted the DNA purifications, cloning, sequence editing and analyses. RV performed most of the statistical analysis and drafted the figures. Both authors read and approved the final manuscript.
